# DeGNServer: Deciphering Genome-Scale Gene Networks through High Performance Reverse Engineering Analysis

**DOI:** 10.1155/2013/856325

**Published:** 2013-11-17

**Authors:** Jun Li, Hairong Wei, Patrick Xuechun Zhao

**Affiliations:** ^1^Bioinformatics Lab, Plant Biology Division, Samuel Roberts Noble Foundation, 2510 Sam Noble Parkway, Ardmore, OK 73401, USA; ^2^School of Forest Resources and Environmental Science, Michigan Technological University, 1400 Townsend Drive, Houghton, MI 49931, USA; ^3^Department of Computer Science, Michigan Technological University, 1400 Townsend Drive, Houghton, MI 49931, USA

## Abstract

Analysis of genome-scale gene networks (GNs) using large-scale gene expression data provides unprecedented opportunities to uncover gene interactions and regulatory networks involved in various biological processes and developmental programs, leading to accelerated discovery of novel knowledge of various biological processes, pathways and systems. The widely used context likelihood of relatedness (CLR) method based on the mutual information (MI) for scoring the similarity of gene pairs is one of the accurate methods currently available for inferring GNs. However, the MI-based reverse engineering method can achieve satisfactory performance only when sample size exceeds one hundred. This in turn limits their applications for GN construction from expression data set with small sample size. We developed a high performance web server, DeGNServer, to reverse engineering and decipher genome-scale networks. It extended the CLR method by integration of different correlation methods that are suitable for analyzing data sets ranging from moderate to large scale such as expression profiles with tens to hundreds of microarray hybridizations, and implemented all analysis algorithms using parallel computing techniques to infer gene-gene association at extraordinary speed. In addition, we integrated the SNBuilder and GeNa algorithms for subnetwork extraction and functional module discovery. DeGNServer is publicly and freely available online.

## 1. Introduction

The advent of high-throughput technologies including microarray experiments and RNA-Seq technologies has generated terabytes of gene expression data for systematically identifying transcriptional regulation and interactions through the reconstruction of gene networks on genome-wide scale. Analysis of whole genome-scale networks can provide a holistic view of all transcription regulations among and within different subnetworks and allows us to gain a more comprehensive understanding of regulation of cellular processes and events. In the past few years, large amount of gene expression data sets from numerous labs has been published and deposited in public databases such as ArrayExpress [1] and Gene expression Omnibus [2], and the volume of this kind of data is still exploding at an accelerated rate. Previous effort in analyzing these public available data has led to the discovery of large amount of novel biological knowledge, making it become increasingly clear that reverse engineering of such “big data” for genome-scale network reconstruction and analysis is one of the most efficient approaches for understanding how life functions through learning holistic transcription regulation and gene interaction.

To date, reverse engineering of aggregated high volume gene expression data for building accurate gene network is still very challenging. The challenge lies in the high dimensionality of gene space and large sample numbers that demand fast and high efficient algorithms, and enhanced computational power as well. A set of the algorithms operates under such a hypothesis that coexpressed [3–5], roughly coordinated genes [6, 7] and genes with dependency [8–10] across a set of samples indicate a functional relationship [11, 12]. As one of the best gene network construction methods, the context likelihood of relatedness (CLR) method [9] utilizing the mutual information (MI) for scoring the similarity of gene pairs has been widely used to decipher gene networks for multiple species, such as yeast, bacteria, mammalian, and plants [9, 13–16]. However, it is computationally infeasible to decipher genome-scale networks for species with large genomes on a single computer due to physical limits on CPU speeds and memory capacities. For example, there are more than thirty-five thousand genes (transcripts) in human genome. To decipher a genome-scale network through such reverse engineering method, it will need to calculate more than 1.2 billion MI values if we evaluate genes in pairwise fashion, and it is more likely that we will need to evaluate genes in triples or quadrants. Even for those species with small genomes, it is still a big computational challenge to use this method. When CLR was used to construct global networks for *Escherichia coli *in [9], the authors had to trim the number of genes down to a few thousands in order to reduce the computational complexity to a manageable scale. Obviously, this kind of gene reduction prior network construction could miss many potential gene regulations and interactions in the constructed networks. This is because many important transcription factors or genes involved in signaling transduction are expressed at low level and do not necessarily have high variability in expression [17–19]. These genes can be easily eliminated during data trimming process.

Meanwhile, the estimation of mutual information adopted in CLR method heavily relies on the number of microarray data sets. The mutual information value could be estimated accurately only when the number of microarray profiles is larger than one hundred [20]. However, as more microarray and RNA-seq data become available in public database, this, in turn, demands fast, accurate, and less computational complexity. Therefore it is urgently called to develop a high performance reverse engineering system for large-scale gene network analysis through both innovations in efficient algorithm development and parallel computing implementation.

In this study, we integrated parallel computing technologies into DeGNServer to accelerate network reconstruction and subnetwork extraction, which enables DeGNServer to analyze the “big data” in at least one hundred times faster than the original mutual information based CLR, making it much feasible for reverse-engineering global gene networks using the data from a large genome and discovering novel biological knowledge. Meanwhile, we integrated multiple gene association methods into our DeGNServer for network construction. The benchmark data set demonstrated that most of these different association-based CLR methods could reach very similar accuracy as the original mutual information-based CLR method. In addition, we also integrated the SNBuilder [21] and GeNa [22] communities-finding algorithms for identifying subnetworks by providing some seed genes. The major purpose of our system is to provide a practical system to construct the gene association networks from large scale gene expression data.

## 2. Implementation

### 2.1. Overview of Gene Network Analysis Methods and Data Analysis Workflow

**Figure 1:**
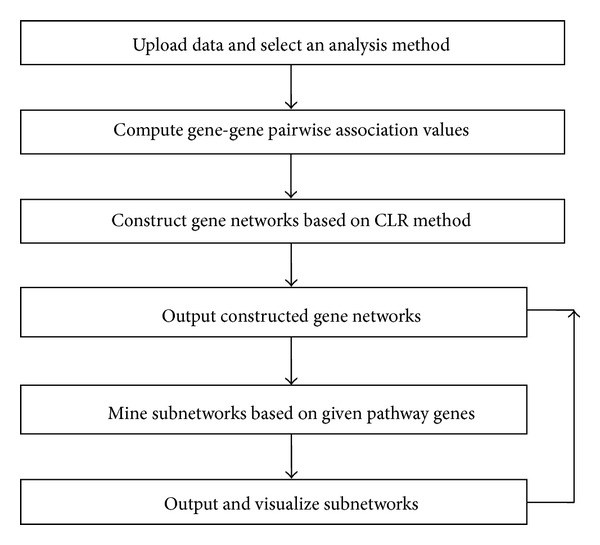
The DeGNServer data analysis workflow.

We extended the CLR method through integrating several gene-gene association estimation methods, of which includes Pearson, Spearman correlation [6], Kendall, Theil-sen [23], and Weighted Rank methods [24] as well as the mutual information-based method proposed in the original CLR method [9], in the DeGNServer. In addition, the recently published method, maximal information coefficient (MIC) method [25], which has demonstrated capability in discovering novel associations in large data sets, was also integrated into our DeGNServer. To help the biologists to interpret the inferred network, we integrated SNBuilder [21] and GeNa [22] approaches for subnetwork analysis/functional module discovery. All algorithms have been implemented and deployed on our in-house parallel computing platform, namely, BioGrid, which has dedicated over 700 CPU Cores.  Figure 1 illustrates the data analysis workflow in DeGNServer. Utilizing our high performance DeGNServer, typical genome-scale gene networks involving 40,000~50,000 gene models could be constructed from expression data that consists of ~200 microarray hybridizations in less than 30 minutes.

### 2.2. Parallel Computing for the Accelerating of GN Construction

To accelerate the GN construction through the parallel computing, we split the whole data sets of these gene pairs into multiple subsets. Let *M* denote the *n* × *m* gene expression matrix, where *n* denotes the number of genes and *m* represents the number of gene expression profiles. The computational complexity of association value for all gene-gene pairs of is *O*(*n* ^2^ × *m*). The reconstruction of network will be very time-consuming when there exists massive number of expression profiles (e.g.,  *n* > 20,000  and  *m* > 1,000). To tackle this issue, we implemented the GN analysis algorithms using parallel computing techniques. When this task is distributed to all the computing nodes in our Biogrid system, the total computational time complexity is then reduced to *O*(*n* ^2^ × *m*/*p*), where *p* is the number of allocated processors.

When a gene regulatory network is inferred from *n* genes, the algorithm will need to compute *n* × (*n* − 1)/2 pairwise associated values. A two-dimensional *n* × *n* matrix D is used to denote these gene pairs. For gene pair (*i*, *j*), the association value of this gene pair will be calculated when the following requirements are satisfied.

(1) When  *n*  is even
  (1) if  i≤⌈n2⌉,  then  j∈[i+1,min⁡(n−1,i−1+⌈n2⌉)],if  i>⌈n2⌉,  then  j∈[i+1,n−1]∪[0,i+1−⌈n2⌉].
(2) When  *n*  is odd
  (2) if  i⌈n2⌉,  then  j∈[i+1,min⁡(n−1, i+⌈n2⌉)],if  i>⌈n2⌉,  then  j∈[i+1,n−1]∪[0,i−1−⌈n2⌉].

For every processor in our Biogrid system, we assign *n*/*p* rows of matrix to this processor for the calculation of their corresponding association values.

### 2.3. Input Detail

DeGNServer accepts normalized expression data either in a tab-delimited text file or tab-delimited text. The server DeGNServer provides two options to construct different networks, that is, the coexpression networks and the CLR method-based association networks. Users may adjust the parameter settings, including gene-gene association estimation method and cut-off threshold, to control the size of constructed networks. After the networks are reconstructed, user may submit a list of genes-of-interest and select different subnetwork identification methods to further mine and visualize the same subnetwork generated from different extraction methods.

### 2.4. Output Detail

DeGNServer lists links to the constructed networks/subnetworks in Cytoscape [26] compatible text files, which can be easily imported into the popular Cytoscape software for downstream analysis. In addition, the DeGNServer output page provides interfaces for query and network visualization through Cytoscape web plug-in [24] for each identified subnetwork.

### 2.5. Technical Detail

**Figure 2:**
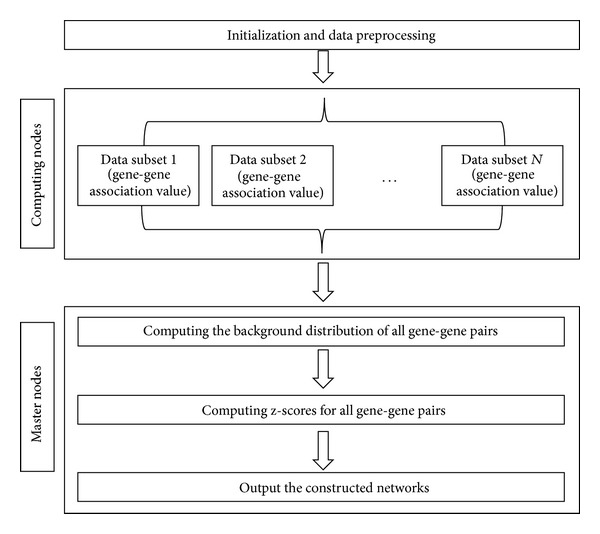
Parallel implementation of the CLR method.

The DeGNServer is currently deployed on Linux using resin Java server 4.0. It has been tested using the popular web browsers, such as Internet Explorer, Firefox, and Google Chrome. The web interfaces are implemented in JAVA and JSP scripts. All backend integrated analysis algorithms are implemented with parallel programming techniques in efficient C++ computing language and are deployed on an in-house developed Linux cluster, namely, BioGrid, which currently consists of about 700 CPU Cores, to achieve high performance computing capacity. Upon job submission through DeGNServer web server, the master node of BioGrid system firstly divides the gene expression matrix into multiple submatrixes and transfers these submatrixes to slave computing nodes in the Linux Cluster. Next, the master node remotely calls to execute the analysis pipelines and monitors analysis progresses in these computing nodes. Finally the master node collects the association values of all gene-gene pairs for gene network construction and subnetwork analysis. For those species with large genomes, the distributions of gene-gene pairs are close to the normal distribution, so we applied the normal distribution to calculate the *z*-score of gene-gene pairs. Based on the preset *z*-score threshold, those gene-gene pairs whose *z*-scores are less than the threshold would be discarded.  Figure 2 illustrates the parallel implementation of the CLR Method.

## 3. Results

### 3.1. Performance Evaluation with Synthetic Data

**Figure 3:**
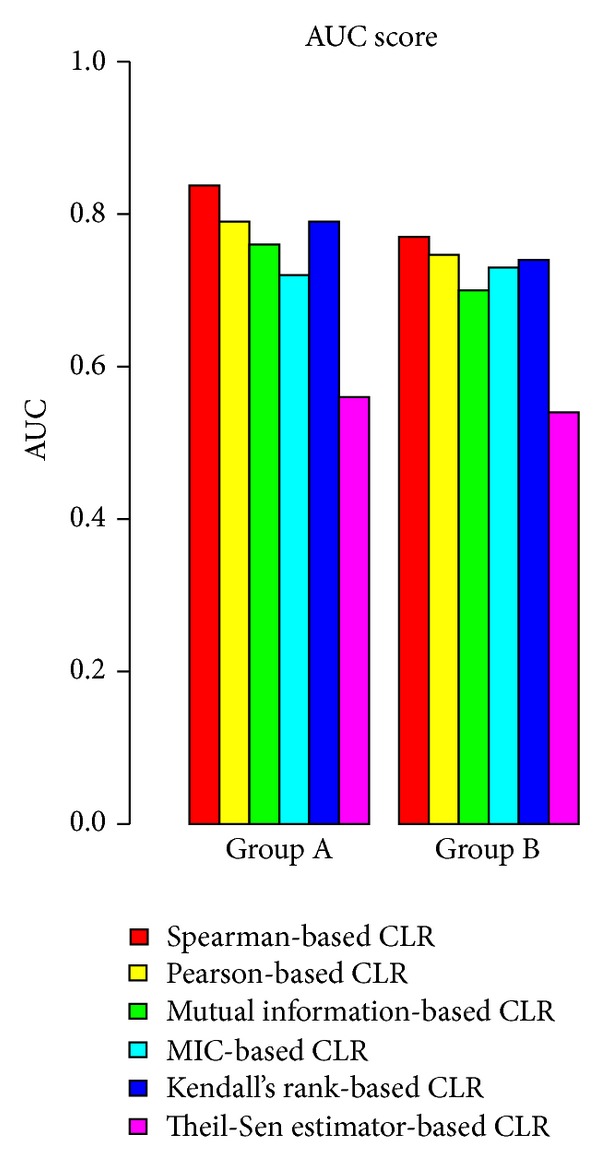
Average AUC scores from different association-based CLR methods for networks with larger and smaller numbers of expression profiles; Group A: networks constructed with smaller number of gene expression samples (30~90 samples), Group B: networks constructed with larger number of expression samples (100~1000). AUC scores were obtained through varying different threhold settings. A perfect model will have AUC score of 1, while random guessing will score an AUC around 0.5.

To comprehensively evaluate performance of integrated network construction methods, we generated two groups of synthetic compendium gene expression data sets, each group with a series of data sets of various sizes, using the SynTReN software [27] and the regulatory network models based upon *Escherichia coli* experimental data as original seeds. The sampled sizes of Group A data sets are 30, 40, 50, 60, 70, 80, and 90, while the sizes for Group B are 100, 200, 300, 400, 500, 600, 700, 800, 900, and 1000 samples. We analyzed each of these compendium data sets with various sample sizes and then generated respective subnetworks containing 50 genes. The prediction accuracy against the corresponding reference network in SynTReN software uses the area under the receiver operating characteristic curve (ROC) curve, namely, the AUC scores [28], to represent the accuracy of each method. The AUC scores resulting from all compendium data sets within each group were averaged, and results of averaged AUC scores for all method in each group are shown in Figure 3.

The ROC curve indicates the change of sensitivity (true positive rate) versus specificity (true negative rate) under different thresholds, and AUC score can represent the accuracy of each method better because it is independent of different thresholds.

The following formula is used to calculate the sensitivity and the specificity:

(3) $$\begin{array}{c} \text{Sensitivity}=\frac{\text{TP}}{\text{TP}+\text{FN}},\\ \text{Specificity}=\frac{\text{FP}}{\text{FP}+\text{TN}}. \end{array}$$

All methods were applied to construct GNs with each sampled data set in either Group A or B with the positive regulatory relationships being counted. We then calculated their respective AUC scores. For each group (smaller and large number of expression data sets), we compared their average AUC scores for different methods. Figure 3 shows that the prediction accuracies of Spearman-based CLR method have higher average AUC scores than other methods, suggesting that Spearman-based CLR method may produce better results in term of network construction.

### 3.2. Case Study 1: Deciphering Genome-Scale Pluripotency Networks in Human Embryonic Stem Cells

#### 3.2.1. Human Stem Cell Microarray Data Set

To validate the performance of DeGNServer, we analyzed genome-scale networks from 189 human stem cell microarray profiles. These data sets were generated in 17 individual experiments in which human embryonic stem cells were treated with various reagents for inducing differentiation. Therefore, this compendium data set is enriched with regulatory events and interaction of pluripotency maintenance and transition from pluripotent stem cells to differentiated cell linages, and thus it can serve as an ideal testing data for the performance of DeGNServer in discovering functionally associated gene subnetworks governing these processes. Of these 189 microarray data sets, there are 104 high-density human gene expression arrays from HG17 assembly. This platform of microarray contains 388,634 probes from 36,494 human locus identifiers. These 104 chips were compiled from 15 experiments in which stem cells were treated with different reagents that disrupted pluripotency. The reagents and the conditions included 12-O-tetradecanoylphorbol-13-acetate (TPA) treatment in conditioned medium, TPA treatment in TeSR medium, BMP4 treatment with FGF, BMP4 treatment without FGF, and coculture with mouse OP9 cells. The remained 85 high-density human gene expression arrays have 381,002 probes from 47,633 human locus identifiers from the HG18. These 85 microarray data sets were compiled from a set of experiments where a variety of different growth factors were applied to human embryonic stem cells at varying conditions for 3 days. Both HG17 and HG18 microarray platforms were manufactured by NimbleGen Systems (http://www.nimblegen.com/). All probes are 60 mers and all chips were hybridized to Cy5 labeled mRNAs extracted from human embryonic stem cells (hESCs) from undifferentiated to differentiated stages. Raw data were extracted using NimbleScan software v2.1. The two data sets were joined by gene mapping via selection of shared common probes between the same genes on the two platforms. More than 99.5% of mapped genes share at least 6 common probes, and the signal intensities from these common probes were normalized with the Robust Multiple-chip Analysis (RMA) algorithm [29]. Thus, the whole data set obtained contains 36,398 genes.

#### 3.2.2. Results on Pluripotency Network Analysis in Human Embryonic Stem Cells

The gene networks including 21,167 genes and 200,000 links were reconstructed in less than 20 minutes with a *z*-score threshold of 4.3 and spearman-based association method. The built network could be retrieved at http://plantgrn.noble.org/DeGNServer/Result.jsp?time4=&sessionid human&method=1_1&cutoff=4.3. We also tested with original mutual information-based CLR, and it took 53.3 hours to complete whole genome-scale network construction.

**Figure 4:**
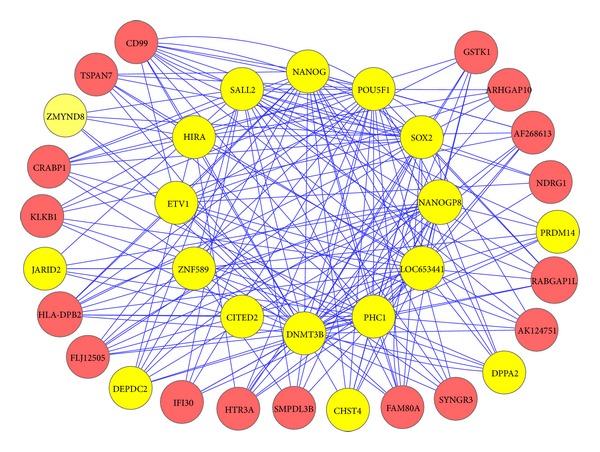
The identified subnetwork contains the essential transcription factors and other genes required for pluripotency maintenance. The twelve genes on the inner ring are transcription factors known to play essential or important role in pluripotency renewal of human embryonic stem cells. These include three master transcription factors, NANOG, POU5F1, and SOX2, which are absolutely required for pluripotency maintenance. The genes located on the outer ring were identified by DeGNServer for being closely coordinated with those transcription factors in the inner ring. The genes on outer ring, but highlighted in yellow, are those that are implicated by the existing literature to participate in the pluripotency renewal. This subnetwork was generated by using SNBuilder method [21] with NANOG, POU5F1, SOX2, and PHC1 as query seeds.

Generally, global networks with huge numbers of regulations and interactions are a “hairball”, from which we can hardly identify any patterns. To facilitate the identification of subnetworks or modules that regulate a specific biological process or developmental program, we integrated both SNBuilder [21] and GeNa [22] methods to extract smaller subnetworks/functional modules by providing a few seed genes. We used NANOG, POU5F1, SOX2, and PHC1 as seed genes to bait the subnetwork shown in Figure 4.

Figure 4 shows the subnetwork that is implicated to control the pluripotency renewal of human embryonic stem cells. The literature evidence supporting the involvement of those transcription factors on inner ring in regulating pluripotency in human stem cells is already shown in our earlier publication and these TFs could be identified by our TF-Cluster that is capable of constructing gene association network with all TFs as an input [6]. However, it cannot be used to build the genome-wide GN mainly due to computational complexity. In this study, our DeGNServer identified 14 of 16 TF genes that were identified previously by TF-Cluster tool from the same data for governing pluripotency renewal. These 14 TFs include three master transcription factors, NANOG, POU5F1 (or OCT4), and SOX2, which are necessary for pluripotency maintenance, and they alone can convert skin cells to induced pluripotent cells [30]. Although two TFs were missed by our method, we identified six more other genes that are to be involved in pluripotency maintenance in human stem cells. In this study, we only examined the existing literature of six genes that are located on the outer rings (Figure 4). The developmental pluripotency-associated 2 (DPPA2) gene plays important roles in the maintenance of pluripotency and proliferation of human embryonic stem cells by regulating chromatin structures [31]. Although there is no direct evidence from human stem cells, study on mouse stem cells shows that DPPA2 knockdown induces the differentiation, while it represses proliferation of mouse embryonic stem cells [31]. PRDM14 is an important determinant of the human embryonic stem cell (ESC) identity, and it works in concert with the core ESC regulators to activate pluripotency-associated genes [32]. PRDM14 binds to silenced genes and serves as a direct repressor of differentiation genes in human stem cells though the exact mechanism of this repressive activity remains unknown. ZMYND8 encodes a zinc finger protein with a complex role in maintaining pluripotency. Although only expressed at low levels, either up- or downregulation of ZMYND8 can induce differentiation in ES cells [33]. JARID2 is a component of chromatin modification complex PRC2 in embryonic stem cells and is required for multilineage differentiation. It plays a role in recruiting PRC1 and RNA Polymerase II to developmental regulators. We found that JARID2 and CD99 in our subnetwork and previous study have shown JARID2 functions together with CD99 in controlling autism spectrum disorder [34]. The exact function of DEPDC2 is currently unknown, but it is known that the promoter of DEPDC2 is bound by the three master transcription factors, NANOG, SOX 2, and POU5F1 as mentioned above [35]. DEPDC2 is a molecular marker for human stem cell [36] though its exact function remains unknown. Similarly, the exact function of CHST4 is currently unknown, but it is know that CHST4 is one of the 16 methylation markers of embryonic stem cells, and these 16 methylation markers also include PRDM14 as mentioned above [37].

To further examine the sensitivity, specificity, and prediction accuracy of the case study described above, we made some assumptions. (1) We assumed that the genes that are evidenced to be involved in pluripotency maintenance in the existing literature are all positive genes; we then counted the true positive (TP) and false positive (FP) genes within each subnetwork. The true negative (TN) and false negative (FN) genes were calculated from the rest of network that was adjusted to the same size of each subnetwork. For comparison, we rescaled all numbers to one hundred before we calculated sensitivity, specificity, and prediction accuracy. The results were shown in Table 1. The results demonstrate the high accuracy of the DeGNServer.

### 3.3. Case Study 2: Deciphering Genome-Scale Pluripotency Networks in Murine Heart Tissues

#### 3.3.1. Mouse Heart Microarray Data Set

We also analyzed a compendium microarray data set from heart tissues of *Mus musculus* to evaluate the efficiency of the DeGNServer. This compendium data set includes 172 Affymetrix microarray chips of platform GPL1261, which contains 45,101 probes. The data was downloaded from NCBI Gene Expression Omnibus (GEO) (http://www.ncbi.nlm.nih.gov/geo/). These 172 microarray data were from nine independent experiments that have the following GEO accession IDs: GSE11291, 15078, 19875, 29145, 30495, 3440, 38754, 5500, and 7781. The compendium data were generated through pooling the raw data of 172 microarray data and then normalized with RMA algorithm [29]. For quality control, we used two methods that were previously described [38].

#### 3.3.2. Overall Performance

The gene networks including 41,742 genes and 3,869,157 links were deciphered in less than 30 minutes with a *z*-score threshold of 3.8 and spearman-based association method. The built network could be retrieved at http://plantgrn.noble.org/DeGNServer/Result.jsp?sessionid=1367625665687&method=1_1&cutoff=3.8#. We also tested with original mutual information-based CLR, which took 81.6 hours to complete whole genome-scale network construction.

#### 3.3.3. Subnetworks Controlling Murine Heart Development

**Figure 5:**
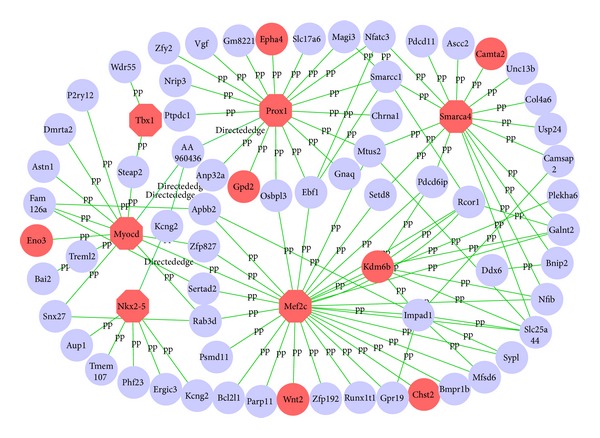
The subnetwork that is responsible for heart growth and development in mouse. The whole genome-scale network was constructed from 175 chips of GPL1261 platform using DeGNServer and then extracted using community-finding algorithm called GeNa [22] with Nkx2-5, Prox1, and Mef2c as query seeds. Genes highlighted in red are implicated by the existing literature to participate in heart growth and development.

The pathway that controls murine heart development can be obtained from NCBI's BioSystems database with an accession number of 672437 [39]. From the pathway diagram, we can find the three central genes, Nkx2-5, Tbx1, and Mef2c, which play very important roles in heart development, as showed up in the subnetwork, we obtained (Figure 5). Nkx2-5 is known to be involved in cardiac muscle cell differentiation [40], proliferation [41], contraction [42], and muscle tissue development [43]. Lack of Nkx2-5 can lead to the myogenic and morphogenetic defects in the heart tubes [43]. Mef2c and Nkx2.5 are known to control common downstream targets and exhibit striking phenotypic similarities when disrupted [43]. Tbx1 affects asymmetric cardiac morphogenesis by regulating Pitx2 in the secondary heart field [44]; it also controls regional coronary artery morphogenesis [45], aorta morphogenesis [46], and blood vessel development [47]. Prox1 is known to function as a direct upstream modifier of Nkx2.5 and is responsible for maintaining muscle structure and growth [48, 49]. CAMTAs promote cardiomyocyte hypertrophy and activate the ANF gene, at least in part, by associating with the cardiac homeodomain protein Nkx2-5 [50]. The transcriptional activity of CAMTAs is governed by association with class II histone deacetylases (HDACs), which negatively regulate cardiac growth [50]. Smarca4, as a nuclear notch signaling component required for the establishment of left-right asymmetry [51], is also essential for heart development by involving chromatin remodeling complexes [51]. Kdm6 interacts with Smarca4 to control T-box family member-dependent gene expression [52]. Wnt2 is required for atrial and inflow tract morphogenesis, and it regulates expansion of secondary heart field progenitors [53]. Myocd controls cardiac muscle cell proliferation, growth, and differentiation [54]. Eno3 is highly expressed in skeletal muscle and heart [55]. The specific function of murine Chst2 is currently unknown, but human umbilical vein endothelial cells predominantly express CHST2 [56, 57]. The heart requires glycerol as an energy substrate through aquaporin 7, a glycerol facilitator [58]. Glycerol is taken into cardiomyocytes and is finally converted to pyruvate by Gpd2 enzymes [59]. *EphA4* mutant mice exhibit defects in the coronal suture and neural crest-mesoderm boundary [60].

### 3.4. Sensitivity, Specificity, and Prediction Accuracy of the above Two Case Studies

To further examine the sensitivity, specificity, and prediction accuracy of the three case studies as shown above, we made some assumptions: (1) for human pluripotency renewal, we assumed that the genes that are evidenced to be involved in pluripotency maintenance in the existing literature are all positive genes; (2) for heart development, due to the large number of genes involved in these biological processes, we cannot search the literature evidence for all genes. We classified all genes involved in heart development to be positive based on gene ontologies. We then counted the true positive (TP) and false positive (FP) genes within each subnetwork. The true negative (TN) and false negative (FN) genes were calculated from the rest of network that was adjusted to the same size of each subnetwork. For comparison, we rescaled all numbers to one hundred before we calculated sensitivity, specificity, and prediction accuracy. The results were shown in Table 1.

## 4. Discussions

We developed the DeGNServer to enable the reconstruction of genome-scale GN using the increasingly accumulated large-scale gene expression data in public domain. Users may use it to generate whole genome scale GNs from large amount of gene expression data in any species. After whole genome GN construction, users can obtain the subnetworks by providing a few genes of interest. All subnetworks generated with different genes of interest and thresholds will be automatically listed online for downloading and studying. When genome-wide network construction was performed with 189 human microarray profiles as an input for DeGNServer, we could identify a subnetwork containing majority of genes involved in pluripotency maintenance in human embryonic stem cells [6, 30, 35]. It is worth mentioning that TF-Cluster pipeline that we developed earlier [6] is capable of building a coordinated network using the same human compendium data set and identifies only those transcription factors located on the inner ring in Figure 4, but it misses all genes that are located on the outer ring in Figure 4 mainly because it can build a local transcription factor coordination network rather than the whole genome-scale network. When genome-wide GNs were constructed using the DeGNServer, we could identify more genes (shown in outer ring in Figure 4) that regulate human pluripotency renewal together with those major transcription factors as shown in inner ring in Figure 4. To test if DeGNServer can identify true subnetworks in different circumstances, we also applied it to a murine compendium data set we downloaded and pooled from GEO database. The data is from heart tissues of *Mus musculus*. We obtained a subnetwork that contains functionally cohesive genes known to control the heart developmental program in mouse. This evidence clearly indicated that the use of DeGNServer can lead to the deciphering of the more comprehensive networks from which we can discover new genes involved in a specific biological process. We thus think that DeGNServer is useful in identifying genes governing a specific biological process, pathway, or a developmental program.

Although we have tested with synthetic data and found that Spearman-based CLR appears to have better performance than any of other methods including original mutual information based CLR, we still make all methods available in DeGNServer. This is because the efficiency of different methods may be dependent on the properties of biological data, as we showed in a previous study [7]. For subnetwork extraction, we integrated both SNBuilder and GeNa algorithms; both are found to be proficient in identifying the true subnetworks. However, GeNa usually produces small subnetworks with cohesive function.

## 5. Conclusions

We have developed a high performance web-based platform, namely, DeGNServer, for genome-scale GN construction and subnetwork extraction. DeGNServer is capable of analyzing gene expression data with very high dimensionality of gene space and very large number of gene expression profiles. As tested, it can analyze hundreds of microarray profiles of human (36,000 genes) for reconstruction of gene association networks within 30 minutes, mainly through the improvement of gene association estimation algorithms and parallel computing in combination. The DeGNServer is as accurate and sensitive as the original CLR method and runs hundreds to thousands times faster. Furthermore, through the integration of network decomposition methods, the DeGNServer is capable of identifying novel functional cohesive subnetworks or modules.

## Figures and Tables

**Table 1:** Sensitivity, specificity, and prediction accuracy of two case studies.

Case studies	TP	FP	TN	FN	Sensitivity	Specificity	Prediction accuracy
Human stem cell	2.42	97.58	99.95	0.05	98%	50.6%	51.2%
Mouse heart	39.6	60.4	97.5	2.50	94.1%	61.7 %	68.6%

Prediction accuracy = ((TP + TN)/(TP + FP + TN + FN)) × 100%.
